# Cost Effectiveness of Voretigene Neparvovec for RPE65-Mediated Inherited Retinal Degeneration in Germany

**DOI:** 10.1167/tvst.9.9.17

**Published:** 2020-08-10

**Authors:** Matthias Fritz Uhrmann, Birgit Lorenz, Christian Gissel

**Affiliations:** 1 Department of Ophthalmology, Justus Liebig University Giessen and University Hospital of Giessen and Marburg, Campus Giessen, Germany; 2 Department of Health Economics, Justus Liebig University Giessen, Giessen, Germany

**Keywords:** RPE65 mutation-associated IRD, Voretigene Neparvovec (Luxturna), quality-adjusted life-years, cost effectiveness

## Abstract

**Purpose:**

Voretigene Neparvovec-rzyl (VN) is the first available treatment for biallelic RPE65 mutation-associated inherited retinal degeneration, which is usually associated with infancy-onset severe visual impairment and complete blindness during the third life decade. We aim to estimate the cost effectiveness of VN in Germany considering medication costs of €410,550 per eye and potential indirect cost offsets by higher labor force participation.

**Methods:**

We developed an individual patient sampling model to simulate patients over their lifetime. In a Monte Carlo analysis, 1000 simulations are performed. Cycle length of the two-state Markov model is 1 year. For each cycle, visual field and best-corrected visual acuity are tracked, compared with natural progression and converted to quality of life. Direct and indirect costs are recorded and the incremental cost-utility ratio is calculated.

**Results:**

In the base case scenario, VN provides 4.82 additional quality-adjusted life-years over a patient's lifetime at an incremental cost-utility ratio of €156,853 per additional quality-adjusted life-year gained. Sensitivity analyses show the robustness of the results when altering treatment effect duration, discounting of quality-adjusted life-years and costs, direct costs, and natural progression.

**Conclusions:**

Under a lifetime perspective, VN proves to be cost effective for the German statutory health insurance system despite high initial treatment costs. Because VN has important implications for future gene therapies, cost-utility analyses have high economic relevance from a societal perspective.

**Translational Relevance:**

Our research analyzes the value of a gene augmentation therapy in clinical care in terms of quality of life gains for patients with blindness from retinal degeneration.

## Introduction

Inherited retinal degenerations (IRDs) can be caused by mutations in more than 300 genes and loci.^1^ Biallelic mutations in the RPE65 gene are associated with an inherited disease referred to as either early-onset severe retinal dystrophy, Leber congenital amaurosis, severe early childhood-onset retinal dystrophy, or retinitis pigmentosa.^2^^–^^7^

Most patients with RPE65-mediated IRD associated with biallelic pathogenic mutations have profound night blindness from birth associated with decreased visual acuity, causing severe visual disability that is further deteriorating early on. Complete blindness typically occurs during the second or third decade of life.^4^

Previously, this disease had been considered as pharmacologically untreatable, and patient care had focused on psychological support and low vision aids. As a novel therapeutic approach, adeno-associated virus–mediated gene augmentation therapy was developed initially in animal models, then in clinical trials.^6^^,^^7^ The drug, voretigene neparvovec-rzyl (VN), was shown to improve visual function in clinical trials by introducing a novel test for functional vision, that is, the multiluminance level mobility test (MLMT). This led to approval for the treatment of confirmed biallelic RPE65-mediated IRDs by the US Food and Drug Administration in 2017 and by the European Medicines Agency in 2018. Recently, the therapy has also been approved by the corresponding authorities in Saudi Arabia and Switzerland. Targeting a specific gene defect, that is, RPE65, aims at the rehabilitation of the visual cycle by supplementing healthy gene copies via viral adeno-associated virus vectors into the retinal pigment epithelium where the gene is expressed. Administered only once by subretinal injection, improvement of rod function is achieved. Beyond the potential of a first treatment for a devastating hereditary retinal disease with early blindness, VN is the first gene therapy for an ocular disease.^8^^,^^9^

## Objective

VN is a novel therapeutic approach and the first treatment option for a previously untreatable retinal disease. VN has a high potential for quality of life (QoL) gains for patients. From a socioeconomic perspective, cost savings can be expected owing to preserved visual ability, including savings on education and pension for the blind and regular participation in the labor market, all of which could contribute to substantial savings in indirect costs. However, the medication cost of €410,550 per eye at administration has to be covered by the German statutory health insurance system. Despite its economic relevance for the German statutory health insurance system, the cost effectiveness of this agent has not been previously evaluated and no socioeconomic model has been developed for Germany yet. To make a decision about the best use of a society's limited health care budget, a cost-effectiveness analysis can inform health care decision makers. We aim to analyze the cost-effectiveness of VN and to set up an individual patient sampling model, which models the therapeutic effects on patients’ health and accompanying economic effects with regard to both direct (e.g., medication cost) and indirect (e.g., labor force participation) costs.

## Methods

Our cost-effectiveness study was conducted from June 3, 2019, to April 27, 2020. An economic model was set up to compare the net costs with the net benefits of VN. We built a probabilistic lifetime model that simulates pathways of treatment-naïve patients with confirmed biallelic RPE65-mediated IRD. Consistent with previously published economic models for RPE65, we simulate 70 patients^10^ in 1000 Monte Carlo simulations,^10^^,^^11^ where parameters are varied by random draws according to their distribution. Patients are equally divided into two groups and chosen by random draws to be treated with VN or with standard of care (SoC). We assume SoC to consist of annual physicians’ checks plus supportive care according to the patient's individual state of visual impairment. Each patient is individually tracked over a lifetime horizon.

The individual patient sampling model uses a two-state Markov framework. The Markov-transition of moving from first state (alive) to second state (dead) is evaluated once in each cycle. Transition probability is a function of age and the sex-specific mortality rate, which is retrieved from German life tables.^12^ Among the patients who are alive, age and health states are tracked for every cycle.

Our health economic model is set up to model a typical cohort of patients suffering from biallelic RPE65-mediated IRD, including age and sex characteristics. The model tracks both the loss of visual function (VF), that is, the area that can be perceived by each eye and the loss of best-corrected visual acuity (BCVA), that is, visual resolution.

The model has a cycle length of 1 year to reflect the disease's slow progression and to be consistent with similar cost-utility analyses in chronic diseases.^11^^,^^13^^,^^14^ VF is measured as the area of the Goldmann III4e isopter and BCVA is measured in logarithm of the minimum angle of resolution. The improvement in functional vision as shown by the MLMT could not be modeled because there are no data on long-term changes of MLMT and no verified link to QoL data available yet.

The model is set up to reflect the core characteristics of the disease. The disease is characterized by profound night blindness from birth associated with reduced visual acuity causing severe visual disability that is further degrading early on. In most patients, total blindness occurs during the second or third decade of life. To reflect these characteristics of the disease, we defined baseline patient characteristics according to the clinical trials’ population.^8^ This includes a mean age of 15.1 ± 10.9 years (range, 4–44 years) with 58% female patients. For the VN group, baseline BCVA is 1.137 ± 0.369 (approximately 0.08 decimals) and baseline VF is 332.9 ± 413.3. For the SoC group, the baseline BCVA is 0.987 ± 0.306 and the baseline VF is 427.1 ± 372.0. Implementation of the phase III clinical trials’ population to an RPE65 simulation model was already successfully performed in similar economic models.^10^^,^^11^^,^^14^

In RPE65-mediated IRD, BCVA, which is often already significantly decreased, is typically relatively stable during the first life decade with a gradual decline starting around life year 15 to 20.^4^ To reflect this natural disease progression, our model assumes a constant BCVA until life year 15 to 20. The model assumes a start of a decrease in the BCVA in life year 15 to 20 and from this point on, a previously validated age coefficient of 0.0436 is used.^14^ For VF, a decrease of −25 sum total degrees per year is assumed, which matches the average natural progression.^4^

**Figure 1.**
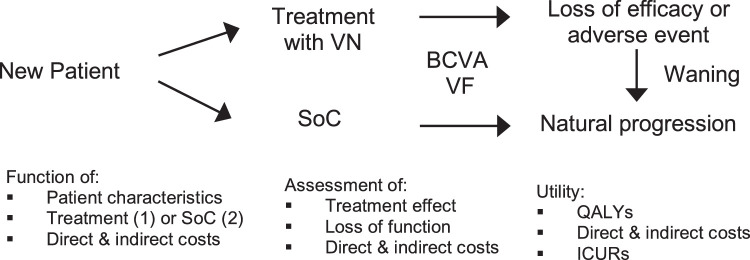
Individual patient sampling model. In the lifetime simulation, each patient is either assigned to the VN group or to the SoC group. During each cycle, BCVA and VF are tracked and direct and indirect costs are assessed. Even in the treatment group, patients can eventually be subject to natural progression depending on the model's assumption toward treatment effect duration. QALYs are recorded and ICURs calculated.

The treatment effect of VN is modeled for all patients in the first cycle based on the results of the phase III clinical trial. This is –0.163 ± 0.336 for BCVA and 302.1 ± 289.6 for VF in the VN group versus −0.312 ± 0.097 for BCVA and –76.7 ± 258.7 for VF in the SoC group. In clinical studies, treatment effects manifested within the first month after application and the effect duration was shown to be constant for 4 years after VN administration.^9^^,^^15^ Our base case scenario assumes that treatment effect is maintained over lifetime, as assumed by Johnson et al.^10^ The sensitivity analysis simulates a treatment effect duration of 10 years to account for uncertainty with regard to the duration of the treatment effect, because no long-term data are available yet. After this period, the effect wanes with an exponential decrease for another 10 years to the level it would have under SoC treatment. This approach is assumed by the Institute for Clinical and Economic Review.^14^ After loss of efficacy or discontinuation owing to an adverse event, patients are treated with SoC (Fig. 1).

After each cycle, health states are compared with natural progression and treatment effect and loss of function are tracked. Quality-adjusted life-years (QALYs) are recorded and the incremental cost-utility ratio (ICUR) is calculated with a linear function, whose validity and reliability has already been described by the Institute for Clinical and Economic Review.^14^

The model simulates patients suffering from RPE65-mediated IRD with and without VN treatment. It simulates the therapeutic effects in terms of visual acuity and visual field. From these parameters, the model estimates a gain in QoL for the patients treated with VN compared with those patients who do not receive the treatment. Multiplying the QoL gains with its duration measured in years results in the computation of a QALY gain, which is a standard measure in health economics. QALYs are often used to assess the benefits of a novel therapy over the existing therapeutic possibilities (in this case: SoC). One QALY is defined as one year of life at full QoL (i.e., QoL = 100 %). It is computed as the product of life years and the corresponding QoL, that is, it can be used to quantify the benefits of QoL improvements over a patient's life span.

To assess the costs of the QALY gains, the model tracks both direct and indirect costs for each patient in each cycle, that is, treatment costs are tracked as well as indirect costs or savings, including cost savings through a higher labor force participation owing to sustained vision. Direct cost calculations include drug costs, fees according to the German Diagnosis-related Group system, and outpatient treatment costs according to German university hospital outpatient reimbursement (Table 1). To reflect a societal perspective in Germany, we adapt the model to reflect indirect costs in proportion to the degree of visual impairment. After each cycle, indirect medical costs, for example, treatment of accidents as a diseases’ result, indirect nonmedical costs, for example, transport or home improvements, and general indirect costs, for example, lost economic productivity, are added. Indirect cost data is implemented from Chuvarayan et al.^16^ (Tables 2A^3^–2C). In the base case scenario, costs are discounted by 3%, as recommended by the German Institute for Quality and Efficiency in Health Care.^17^ This process is consistent with previously published economic models for the German statutory health insurance system.^18^^,^^19^

**Table 1.** Direct Costs

Entry	€
Voretigene-Neparvovec per eye	410,550
Pharmacy per eye	2000
DRG G18Z reimbursement per eye	2840
Outpatient reimbursement per quarter	220
Direct costs year 1	831,660
Direct costs year *n*	220

Direct costs per year are added for the treatment of both eyes. DRG, diagnosis related group.

**Table 2A.** Indirect Medical Costs

BCVA	€/6 Month	SD
<0.02	5116	9938
0.05–0.02	3342	4854
0.3–0.05	2400	5483

SD, standard deviation.

Source: Chuvarayan et al.^16^

**Table 2B.** Indirect Nonmedical Costs

BCVA	€/6 Month	SD
<0.02	10,868	115,022
0.05–0.02	3940	21,011
0.3–0.05	1207	6442

SD, standard deviation.

Source: Chuvarayan et al.^16^

**Table 2C.** General Indirect Costs

BCVA	€/6 Month	SD
<0.02	3948	7437
0.05–0.02	3934	7892
0.3–0.05	3061	6891

SD, standard deviation.

Source: Chuvarayan et al.^16^

To follow the German Institute for Quality and Efficiency in Health Care guidelines for the sensitivity analysis, we change discounting for costs and QALYs to 0%. To evaluate the direct costs’ impact on the result, we evaluate a ±10% change in direct costs in additional sensitivity analyses. The purpose of the sensitivity analyses is to show the effects of slight changes in underlying assumptions on the overall health economic result, for example, costs could be over- or underestimated or the valuation of future costs or health gains might vary (i.e., their discounting factor for the evaluation of their present value).

## Results

**Figure 2.**
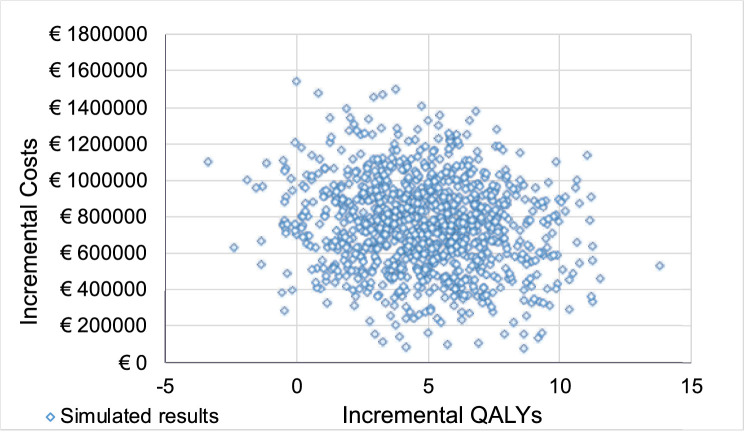
Cost-effectiveness plane. Additional costs and QALYs gained for 1000 simulations in the base case. Each *blue dot* shows the result of one of 1000 simulations with incremental QALYs gained over SoC on the *x*-axis and additional costs incurred over SoC on the *y*-axis.

**Figure 3.**
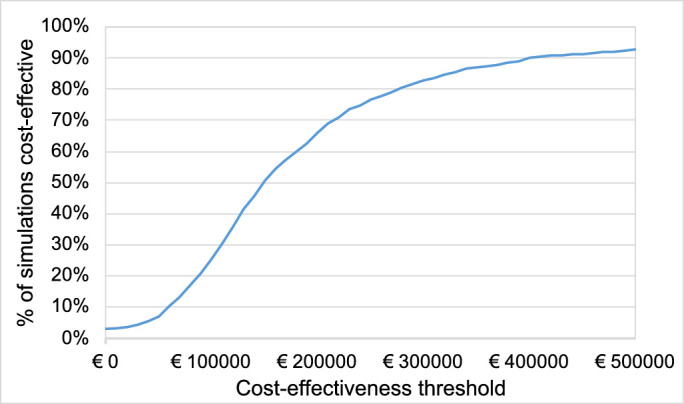
Cost-effectiveness acceptability curve. Percentage of 1000 simulations in the base case, which would be deemed cost effective under the respective cost-effectiveness threshold. Once the simulations’ ICUR is lower than the threshold, the simulation is considered as cost effective. The *blue curve* shows which percentage of 1000 simulations would be deemed cost-effective under the threshold on the *x*-axis.

In our base case scenario, patients treated with VN gain more QALYs at higher total costs than patients in the SoC group; that is, patients benefit clinically from the therapy but the benefit comes at a cost. On average, VN provides 4.82 additional QALYs for each patient over their lifetime; that is, on average, the clinical benefit from VN therapy corresponds to a gain of 4.82 life years at 100% QoL for each patient. Additional overall costs for VN are €755,566. The ICUR per additional QALY gained is €156,853; that is, each life-year gained with 100% QoL incurs costs of €156,853. The results of 1000 Monte Carlo simulations are plotted on the cost-effectiveness plain to display the variance of both clinical and economic outcomes (Fig. 2). A cost-effectiveness–acceptability curve is calculated to visualize the percentage of cases that would be cost effective for a specific cost threshold per additional QALY gained (Fig. 3).

**Figure 4.**
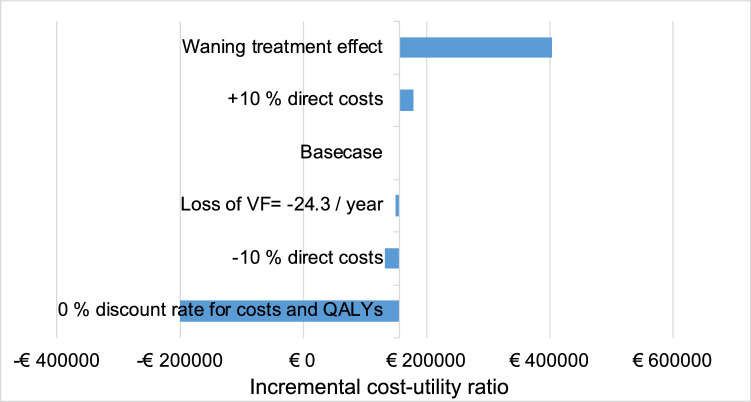
Sensitivity analysis' results. For each sensitivity analysis, the ICUR is recorded and presented in the *blue bar* compared with the base case value. A bar to the left indicates a lower ICUR, that is, a more favorable cost-utility ratio. The *y*-axis is centered on the base case's ICUR.

In our sensitivity analysis, we estimate a treatment effect duration of 10 years, followed by a waning period with an exponential decline in BCVA and VF to SoC levels. This decreases additional QALYs comparing VN with SoC to 2.89 QALYs. Additional overall costs increase to €1,169,403 and the ICUR to €404,774. This sensitivity analysis emphasizes the importance of treatment effect duration for the health economic outcome; that is, the favorable result of the base case analysis depends on a lasting effect of VN therapy. The greatest decrease of ICURs can be seen by a 0% discount rate on costs and QALYs. This leads to a negative ICUR of –€199,700, that is, patients gain more QALYs at lower total costs. Altering direct costs +10% and −10% at 3% cost discounting shows a direct correlation with the ICUR increasing to €179,852, and decreasing to €134,056. Altering the diseases’ natural progression for VF loss shows a robust ICUR of €151,238. Results of our sensitivity analysis and parameters’ influence on ICUR are presented in Figure 4.

## Discussion

The health economic analysis of VN raises three important points. First, our analysis has shown that its cost effectiveness is not trivial. It depends not only on its upfront medication cost, but also on indirect cost offsets, for example, smaller productivity losses owing to higher labor force participation. Furthermore, the therapy's special status as a breakthrough therapeutic concept needs to be considered. Second, the analysis presented in this article was conducted for the German market and needs to be compared with analyses for other health care systems. Third, our sensitivity analyses warrant a discussion of the determinants of the therapy's cost effectiveness.

In our analysis, we modeled the IRD as a congenital disease with early onset severe vision impairment, usually leading to total blindness by the third decade of life and an inability to participate in the labor force, that is, a loss of QoL and a loss of productivity for decades.

Despite the potential clinical benefits and improvements of patients’ QoL owing to therapy with VN, medication costs of €410,550 per eye up front raise questions about the affordability of such a therapy from a societal perspective. Our analysis shows that patients benefit from substantial gains in QoL and QALYs. The medication costs are partly offset by savings in indirect costs, which can be attributed to the beneficial effects of the therapy. In addition, the unique status of VN being both the first gene therapy and the only treatment option for an ultrarare disease may have important implications for future gene therapies. Pharmacoeconomic evaluation has to consider both high direct costs at application and long-term benefits, because moderate to severe visual impairment causes overall annual costs of €49.6 billion in Germany.^16^

Despite VN's economic relevance, our model is the first one to assess VN's cost effectiveness for the German statutory health insurance system. To identify further literature, we conducted a PubMed search on April 27, 2020, for “cost voretigene neparvovec.” Out of eight hits, three cost-effectiveness studies were identified. Two studies were found for the United States and one study for the UK. Because indirect cost data vary greatly between different countries, a direct comparison between studies is not possible. However, additional QALYs gained and ICURs of our results point in the same direction as the 2020 study by Viriato et al.,^11^ who estimated VN to be cost effective for the UK and assumed a gain of 6.4 QALYs. This study confirms our finding that substantial QALY gains can be attributed to the therapy when its effects are modeled with a life time perspective.

In 2019, Johnson et al.^10^ suggested VN to be cost effective in the United States but, in contrast, the Institute for Clinical and Economic Review^14^ determined in their 2019 simulation model VN unlikely to reach cost effectiveness in a US context. Johnson et al.^10^ mentioned a lack of patient-level data and of RPE65-specific health utilities and the use of inappropriate indirect costs in the approach of the Institute for Clinical and Economic Review.^14^ For a cost-utility analysis, the lack of indirect cost data is challenging, because samples of most studies focus on age-related visual impairment,^16^ which complicates indirect cost assessment. Although no correlation is found between cost of visual impairment and its primary cause,^16^ age plays a key role in the estimation of indirect costs over a patient's lifetime. Hence, we use a study with younger patients on average.^16^

In addition, we are able to use natural history data for RPE65-mediated IRD^4^ in our calculations. For example, this includes a natural disease progression with a constant BCVA in the first life decade and a gradual decline to start afterwards. Other studies do not address this specific characteristic of BCVA in RPE65-mediated IRD. For VF, we assume a linear decrease of −25 sum total degrees^4^ to meet natural progression, whereas the Institute for Clinical and Economic Review^14^ implemented a decrease of −24.3 per year. In our sensitivity analysis, we analyze this approach and we demonstrate the robustness of the results.

Our model is limited by sparse utility data, which is common for RPE65 cost-utility analyses. All existing models only use BCVA and VF to define health states and to calculate utility. The clinical trial for VN showed significant improvements in VF, but uses the enhancement of MLMT scores as the primary outcome measure, which measures the ability to navigate in low-to-moderate light. In addition, an increase in the full-field stimulus threshold test was observed. Improvements of average BCVA were found in the end points for 20 patients in the VN group compared with 9 patients in the control group, but were not statistically significant.^8^ The enhancement of MLMT indicates an important improvement for everyday activities resulting from less dependence of the functional vision on varying light levels, thus improving independent mobility. Additional QALY gains as a consequence of this enhanced functional vision would be likely. However, because the MLMT is a novel primary end point, there is no verified link to QoL data and no data on long-term changes of MLMT are available yet, which leaves uncertainty around comprehensive pharmacoeconomic outcomes. Given that there are significant improvements in both MLMT and full-field light sensitivity test, which are not captured by our model, the QoL gains are likely underestimated. Our model may be a conservative estimation of VN's benefits, as the incorporation of a wider array of clinical benefits would improve VN's ICUR.

In our analysis, the ICUR and indirect cost offsets are driven by the duration of treatment effect. Comparing the assumption of a decreasing treatment effect versus a lifetime treatment effect in our sensitivity analysis, a loss of incremental QALYs and an increase of costs can be observed. A decreasing treatment effect is mainly associated with a loss of QALYs and higher overall costs, primarily driven by the increase of indirect costs. This is consistent with the results of Johnson et al.^10^ and Viriato et al.,^11^ where the treatment effect was also identified as the largest individual driver. We expect that estimation of treatment effect duration may be one of the main factors for cost-utility analyses not only in VN, but also in many future gene therapies. Although not yet proven, a long-term benefit of gene therapies is subject to discussion as long-term gene expression may be induced by virus-mediated gene vectors.^20^

In our sensitivity analysis, we changed discounting to 0% as recommended by the German Institute for Quality and Efficiency in Health Care.^17^ This change leads to a negative ICUR, that is, higher QALYs at lower total costs for VN therapy, which is similar to the base case results of Johnson et al.^10^

For our base case analysis of VN therapy, we calculated an ICUR of €156,853 per additional QALY gained. For Germany, no ICUR threshold has been defined. In the UK, the National Institute for Health and Care Excellence uses for its Highly Specialised Technology processes a threshold of £100,000 per QALY. However, for treatments with a gain of 10 to 30 undiscounted QALYs, a weighting between 1 and 3 may be applied and a threshold up to £300,000 per QALY is assumed to be cost effective.^21^ VN was recommended by the National Institute for Health and Care Excellence in October 2019.^22^ In the United States, a threshold of $150,000 per QALY for nonrare and $250,000 per QALY for ultra-rare conditions may be assumed as the willingness-to-pay increases for treatments of rare diseases.^14^^,^^23^ Given the small patient population, VN's costs generally are very likely to be covered.

Our sensitivity analysis shows that the ICUR is directly driven by VN's direct costs and rebates would lower the ICUR significantly. In the UK, a confidential discount on the price list has been agreed upon.^11^ For Germany, price negotiations of VN may have a direct impact on pricing of future gene therapies and on their cost effectiveness. Our cost-utility analysis shows that VN is likely to be cost effective and may be beneficial not only to patients but also from a societal perspective in Germany. Further opportunities might arise by using VN in younger patients which would probably lower indirect costs owing to better preserved visual ability.
